# Treatment-resistant venous thrombosis and pulmonary embolism in a patient with granulomatosis with polyangiitis: a case report

**DOI:** 10.1186/s40981-021-00478-0

**Published:** 2021-10-02

**Authors:** Chiharu Wakuda, Yoshitaka Aoki, Sho Sugimura, Takayuki Katsuragawa, Yukako Obata, Soichiro Mimuro, Matsuyuki Doi, Yoshiki Nakajima

**Affiliations:** Department of Anesthesiology and Intensive Care Medicine, Hamamatsu University School of Medicine, 1-20-1 Handayama, Higashi-Ku, Hamamatsu, Shizuoka 431-3192 Japan

**Keywords:** Granulomatosis with polyangiitis, Venous thrombosis, Pulmonary embolism, Vena cava filters, Case report

## Abstract

**Background:**

We herein present a case of venous thrombosis that developed more than 20 years after diagnosis of granulomatosis with polyangiitis (GPA), although many reports of GPA have described venous thrombosis within 1 year of diagnosis.

**Case presentation:**

A 73-year-old man with GPA was admitted for lower extremity swelling and diagnosed with venous thrombosis and pulmonary embolism. On the second day, catheter-based thrombolysis was unsuccessful, and inferior vena cava filter insertion and anticoagulation were performed. On the third day, respiratory disturbance and loss of consciousness appeared and progressed. The patient died on the fifth day. The autopsy revealed a large thrombus in the inferior vena cava filter, and death of progressive venous thrombosis was suspected.

**Conclusions:**

We experienced a case of venous thrombosis that developed 20 years after diagnosis of GPA, although GPA is frequently associated with venous thrombosis immediately after diagnosis. The thrombosis progressed rapidly and was resistant to treatment.

## Background

Granulomatosis with polyangiitis (GPA) is a type of anti-neutrophil cytoplasmic antibody (ANCA)-associated vasculitis, previously known as Wegener’s granulomatosis. The incidence of venous thrombosis in patients with GPA is reportedly higher than that in the general population and in patients with systematic lupus erythematosus and rheumatoid arthritis [1]. Venous thrombosis associated with GPA is more common in the active phase, especially within the first year after the diagnosis of GPA [2]. However, few reports have described rapid progression of venous thrombosis as a late complication of GPA.

We herein describe a patient who developed pulmonary embolism from venous thrombosis more than 20 years after the diagnosis of GPA. Despite appropriate treatment in the intensive care unit, including endovascular and anticoagulation therapy, the patient’s symptoms progressed and he died 5 days after admission.

## Case presentation

A 73-year-old man (body weight, 72.2 kg; height, 158 cm) had been diagnosed with GPA 20 years previously based on optic narrowing, upper respiratory symptoms, multiple nodules in the lungs, and positive proteinase 3 (PR3)-ANCA (41 U/mL) and was treated with prednisolone and methotrexate; he did not receive heparin therapy. His PR3-ANCA level was 48.9 U/mL 2 years before admission to our hospital, 53.6 U/mL 6 months before admission, and 83.8 U/mL immediately before admission; despite this increasing trend, however, he showed no subjective symptoms. Before admission, he was being treated with prednisolone (13 mg/day), methotrexate (8 mg/day), and azathioprine (25 mg/day). His medical history otherwise included coronary angina pectoris, hypertension, type 2 diabetes mellitus, Hashimoto’s disease, benign prostatic hyperplasia, thoracolumbar compression fracture associated with osteoporosis, and glaucoma.

**Fig. 1:**
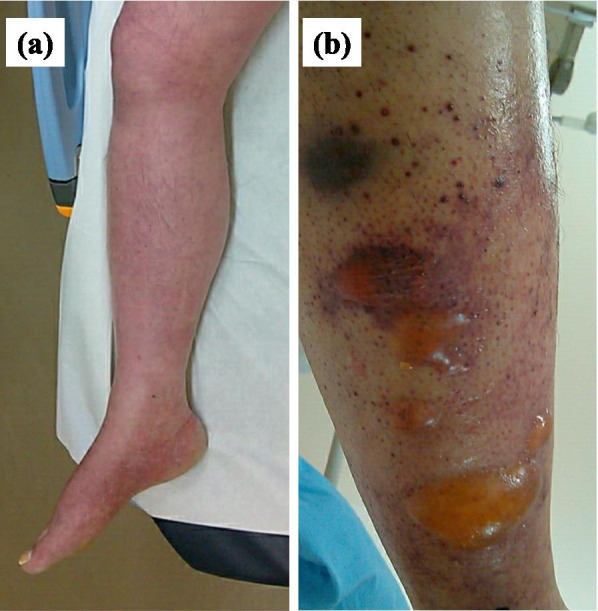
Clinical appearance of the right lower leg. **a** Redness and edema were present on day 1. **b** The redness and edema worsened with blister formation on day 4

**Fig. 2:**
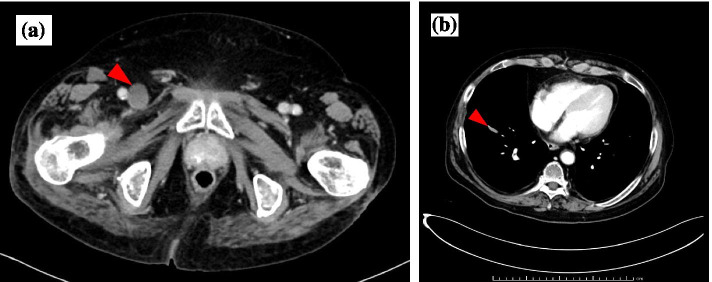
Computed tomographic images. Computed tomography revealed **a** thrombosis in the common iliac vein, causing dilation and occlusion of the vein, and **b** micro-thromboembolism in the pulmonary artery within the right lower lobe

On day 1, the patient was admitted to the hospital for right lower extremity pain. Physical examination revealed muscle weakness and edema in the right lower leg, resulting in emergency admission (Fig. 1a). Computed tomography showed a thrombus in the right common iliac vein (Fig. 2a) and a micro-thromboembolism in the right pulmonary artery within the right inferior lobe (Fig. 2b). His PR3-ANCA level was elevated before admission, but acute progression of GPA was not suspected based on his physical examination findings and computed tomography images. The patient was diagnosed with pulmonary embolism due to deep vein thrombosis and began oral treatment with rivaroxaban (30 mg/day). His creatine kinase level (26 U/L) and creatinine level (0.77 mg/dL) were not elevated. However, his soluble fibrin and D-dimer levels were elevated due to the venous thrombosis (Table 1).

**Table 1:** Changes in markers of coagulation and fibrinolysis

	Entering a hospital (Day 1)	Day 2	Entering the intensive care unit (Day 2)	Day 3	Day 4	Day 5
Platelets (×10,000/μl)	13.4	14	10.4	8.3	6.8	4.8
Soluble fibrin (μg/ml)	61.1	41.7	20.1	10.8	16.6	19.2
D-dimer (μg/ml)	14	14.6	5.8	3.3	2.6	1.2
Prothrombin time (s)	11.7	20.5	39.3	51.4	30.5	23.8
Activated partial thromboplastin time (s)	23.9	31.6	50.7	>200	>200	>200
Fibrinogen (mg/dl)	311	350	265	273	398	392
Antithrombins (%)	96	-	98	91	54	61
Thrombin–antithrombin complex (ng/mL)	23.6	-	-	-	-	-
Plasmin–α2-plasmin inhibitor complex (μg/mL)	6.5	-	-	-	-	-
Protein S antigen level (%)	100	-	-	-	-	-
Protein C antigen level (%)	98	-	-	-	-	-

On day 2, the patient’s leg pain and motor deficits worsened, and his creatine kinase level (435 U/L) and creatinine level (2.03 mg/dL) became elevated. Therefore, urgent inferior vena cava filter placement and catheter-based thrombolysis were performed. Interventional radiology techniques allowed an inferior vena cava filter to establish in the inferior renal vein approaching the right internal jugular vein. As the catheter for thrombolysis was about to be placed through the right lower extremity, pulseless ventricular tachycardia suddenly occurred, and cardiopulmonary resuscitation was performed. The patient’s heartbeat resumed, but the surgery was aborted and he was admitted to the intensive care unit on an emergency basis. Tracheal intubation was not performed at this time because his level of consciousness had improved entirely. The cause of the intraoperative ventricular tachycardia was hyperkalemia (6.3 mmol/L) with myonephropathic metabolic syndrome due to severe reflux to the right lower extremity secondary to the venous thrombosis. Continuous renal replacement therapy was started to treat the acute kidney injury and hyperkalemia, and heparin therapy was started for the thrombus. The medical team discussed whether to perform a right thigh amputation, but surgery was considered contraindicated because of the patient’s poor general condition. Additionally, the patient began treatment with continuous noradrenaline administration for hypotension, albumin preparation administration for hypoalbuminemia, and thrombomodulin alpha for disseminated intravascular coagulation syndrome.

Anticoagulation with heparin prolonged the activated partial thromboplastin time to more than 200 seconds after day 3 (Table 1), but the patient’s symptoms gradually worsened. On the morning of day 4, noninvasive positive-pressure ventilation was performed for hypoxemia, but the patient became progressively unconscious and unable to speak in the afternoon. His right lower extremity showed obvious blistering (Fig. 1b). His soluble fibrin level decreased once with anticoagulation therapy but subsequently increased again (Table 1). He also showed an increased fibrinogen level, decreased antithrombin level, and decreased platelet count, suggesting a hypercoagulable state. On day 5, after discussion with the family, it was decided not to attempt to operate or resuscitate the patient. The patient died on day 5.

**Fig. 3:**
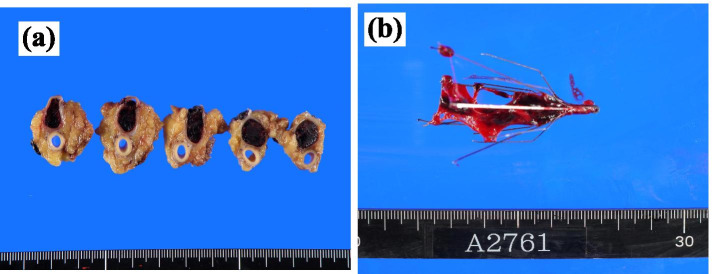
Pathological specimen of **a** right femoral vein and **b** inferior vena cava filter. Note the embolism and occlusion in the right femoral vein and inferior vena cava, in contrast to the intact femoral artery

After the patient’s death, his family provided written consent for pathological autopsy and publication of the present case report. The pathological autopsy showed no thrombus in the femoral artery, but it revealed severe stenosis and occlusion from the right common iliac vein to the junction of the right and left iliac veins (Fig. 3a) and a thrombus in the inferior vena cava filter (Fig. 3b). There was no GPA-associated granulomatous inflammation with necrosis, necrotizing vasculitis, or necrotizing glomerulonephritis. Additionally, there were no tumors that could cause venous thrombosis.

## Discussion

We experienced a case in which a patient with GPA developed venous thrombosis and pulmonary embolism as late complications. His condition progressed and he died despite anticoagulation and insertion of an inferior vena cava filter. The notable characteristics of this case are that venous thrombosis and pulmonary embolism developed late in the course of GPA and that the disease progressed rapidly regardless of treatment.

In this case, venous thrombosis and pulmonary embolism occurred more than 20 years after the diagnosis of GPA, which is very unusual. We suspected sepsis, cancer-related thrombosis (Trousseau’s syndrome), heparin-induced thrombocytopenia, or antiphospholipid antibody syndrome as the differential diagnoses of acquired hypercoagulability and decreased fibrinolysis; however, the imaging and blood test results were not supportive of these diagnoses. Therefore, we considered delayed complications of GPA as a diagnosis of exclusion. GPA is reportedly associated with a high incidence of venous thrombosis (8–18%) [1–5]. However, the incidence of venous thrombosis is higher in the active phase of the disease early after GPA diagnosis. Stassen et al. [4] reported that the incidence of venous thrombosis during the active disease stage was 6.7 per 100 person-years, and that during the inactive disease stage was 1.0 per 100 person-years (*P* < 0.0001). Additionally, Liapi et al. [6] reported that the incidence decreased over time from diagnosis: 20.4 per 100 person-years by 3 months post-diagnosis, 8.9 per 100 person-years at 4 to 6 months, and 1.5 per 100 person-years at 7 to 12 months. Based on our report, venous thrombosis and pulmonary embolism should be kept in mind as possible late complications of GPA, even in the absence of other symptoms of GPA.

The second notable characteristic of this case is the rapid progression of venous thrombosis. We initially suspected that the rapid progression of consciousness impairment was due to GPA-induced cerebral neuropathy and that the progression of renal failure was due to GPA-induced glomerulonephritis. However, the pathological autopsy revealed no apparent lesions in the brain or kidney. Therefore, we concluded that this case purely involved progression of a treatment-resistant thrombus. The patient’s plasma levels of protein C and protein S were normal. His plasma antithrombin level was relatively well maintained until death, suggesting that he had not developed severe endothelial damage that could have caused abnormal vascular permeability. Additionally, his activated partial thromboplastin time was markedly prolonged, indicating a sufficient pharmacological effect of heparin. Despite this anticoagulant state, the plasma soluble fibrin concentration (which directly reflects hypercoagulability) remained abnormally high, while the D-dimer concentration (which indicates fibrinolytic activity) decreased to near normal. The laboratory data showed that coagulation was abnormally enhanced while fibrinolysis was suppressed. The concentration of plasma fibrinogen, one of the acute-phase reactants induced by inflammation, remained high, suggesting that plasminogen activator inhibitor-1 was also induced as an acute-phase reactant. The degree of fibrinolysis suppression was strong, and a gene polymorphism may have induced the development of a large amount of plasminogen activator inhibitor-1 by stimuli such as inflammation. The cause of the increased risk of venous thrombosis in patients with ANCA-associated vasculitis, especially when the disease is active, is unknown. Hilhorst et al. [7] reported that patients with ANCA-associated vasculitis in remission are in a more coagulable state than healthy controls and that elevated factor VIII levels measured in these patients suggest persistent endothelial activation and dysfunction. Berden et al. [8] reported that 24% of patients with ANCA-associated vasculitis had anti-plasminogen antibodies, 18% had anti-tissue plasminogen activator antibodies, and tissue plasminogen activator alterations were more common in patients with anti-plasminogen antibodies. Therefore, altered endothelial function, increased coagulability, and decreased fibrinolysis may increase the risk of venous thrombosis.

## Conclusion

We experienced a case of venous thrombosis and pulmonary embolism that occurred 20 years after GPA onset. The venous thrombosis did not respond to treatment, the condition progressed, and the patient died. Venous thrombosis associated with GPA often occurs immediately after diagnosis, but the onset in the present case occurred during the remission phase. Hypercoagulability and decreased fibrinolysis in patients with GPA in remission may contribute to the rapid progression of treatment-resistant venous thrombosis.

## Data Availability

The datasets used in the current study are available from the corresponding author on reasonable request.

## Abbreviations

GPA: Granulomatosis with polyangiitis
ANCA: Anti-neutrophil cytoplasmic autoantibody
PR3: Proteinase 3
